# Factor XI and XII inhibitors–Dawn of a new era

**DOI:** 10.1016/j.ihj.2025.02.007

**Published:** 2025-02-25

**Authors:** Chhabi Satpathy, Trinath Kumar Mishra, Anshu Kumar Jha

**Affiliations:** Department of Cardiology, MKCG Medical College and Hospital, Berhampur, Odisha, India

**Keywords:** Coagulation pathway, Factor XI inhibitor, Factor XII inhibitor, Haemostasis

## Abstract

The history of coagulation cascade dates back to 17th century. The extrinsic and intrinsic pathways were proposed in 1998. Extrinsic pathway includes the tissue factor and stable factor which activates factor X and with help of factor V, this converts prothrombin to thrombin which is stabilised by factor XIII. This helps to seal the bleeding vessel and is a physiological process as there is only “limited” production of thrombin which doe not expand beyond the damaged site due to absence of tissue factor. On the other hand intrinsic pathway is activated by polyanions, neutrophilic extracellular traps which are present during infection and inflammation. These activate factor XI which activates factor X with the help of factor IX and VIII and then the common pathway ensues. But newer discoveries have shown that this is a very simplified way of explaining the coagulation system. The researches propose that haemostasis is divided into initiation, amplification and propagation phase. Also, the factor VII-tissue factor complex formed activates factor IX and leads to sustained thrombin production as the amount of thrombin produced by extrinsic pathway alone is not sufficient to form a haemostatic plug. Thrombin also activates factor XI and lead to self perpetuation of intrinsic pathway.

All the anticoagulants have an inherent property of bleeding. So the newer factor XI and XII inhibitors focus to inhibit the excessive thrombin production without hampering the physiological haemostasis process. This is supported by the fact that congenital factor XI and XII deficiency does not cause excessive bleeding but increased levels did make patients more vulnerable to thromboembolism.

This review shall focus on the various factor XI and XII inhibitors which are in the pipeline.

## 1 Introduction

### 1.1 Coagulation cascade–classical concept

**Fig. 1:**
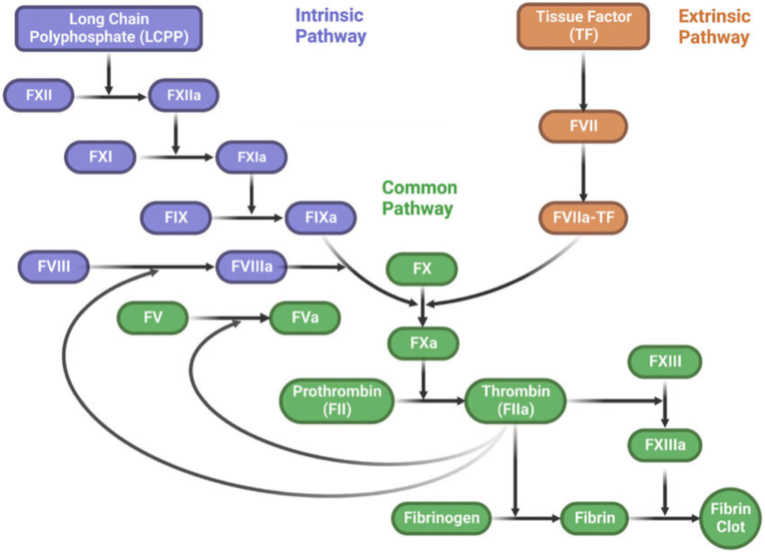
The classical concept of coagulation pathway. F-factor, NETs-Neutrophil Extracellular Traps.^10^.

Human body is a marvellous creation of nature with various complex mechanisms simultaneously occurring to keep it in an equilibrium state. One of which is the coagulation - anticoagulation system. History dates back to 17th century when Malphigi separated red blood cell and serum and showed fibre formation using his single lens microscope.^1^ Since then as science progressed, the coagulation pathway also kept on changing and what we read today about extrinsic and intrinsic pathway was deciphered in 1998.^2^ Extrinsic pathway, also known as tissue factor pathway, is initiated when stable factor/proconvertin (factor VII), comes in contact with factor III (tissue factor - TF). This activates factor X (Stuart Prower factor). Activated factor X in the presence of factor V (Labile factor/Proacclerin), calcium, tissue and platelet phospholipid forms the prothrombinase complex. This complex converts factor II i.e. prothrombin to thrombin which further cleaves circulating fibrinogen i.e. factor 1 to insoluble fibrin monomers which further changes to fibrin polymer and activates factor XIII i.e. fibrin stabilising factor. This fibrin stabilising factor crosslinks fibrin polymers which are incorporated in the platelet plug.^3^ This creates a fibrin mesh which stabilises the clot and forms a haemostatic plug and seals the bleeding. This is a physiological process which is initiated whenever there is bleeding from an injured vessel and is known as “haemostasis”. Here, there is “limited” production of thrombin, sufficient enough to form a haemostatic plug to stop bleeding. Limiting the haemostatic plug expansion beyond the damaged site occurs due to absence of tissue factor in the neighbouring normal areas.^4^ Tissue factor, which is present in sub endothelium is not activated until that layer is exposed.^5^ Parallel to it runs an intrinsic pathway, also known as, contact pathway which is initiated by activation of factor XII (Hageman factor) by polyanions, polyphosphates and neutrophilic extracellular traps or medical devices.^6^^,^^7^ These factors are present during infection and inflammations. This leads to activation of plasma thromboplastin antecedent factor (factor XI). Activated factor XI further activates factor IX (Antihaemophilic factor B or Christmas factor) and this along with its cofactor (factor VIII - Antihaemophilic factor A) forms a complex on a phospholipid surface and activate factor X. From this point onwards, the common pathway ensues as described in the extrinsic pathway above.^3^ This pathway was initially not given much of an importance as the deficiencies of factor XII or XI did not give rise to significant bleeding.^8^^,^^9^ So it was thought that contact pathway has little role *in vivo* and only had a role *in vitro* in labs when blood came in contact with negatively charged surfaces. Fig. 1^10^

### 1.2 Coagulation pathway–newer concept

**Fig. 2:**
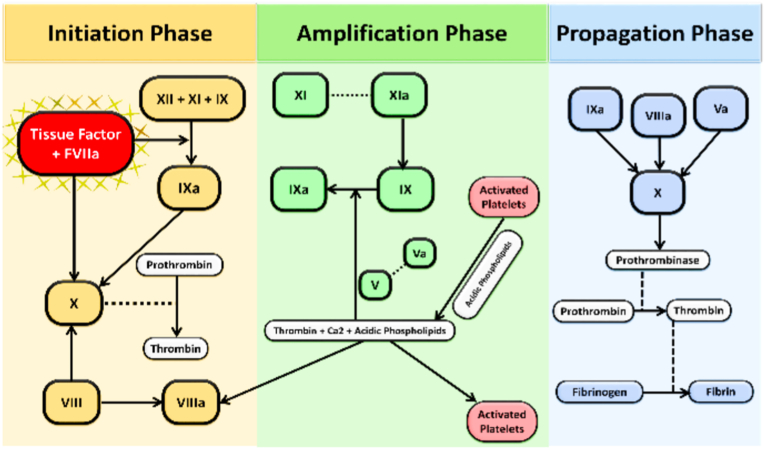
The new concept of coagulation pathway^12^.

With increasing knowledge of the coagulation pathway it became clear that labelling these two pathways as a completely different entity running in parallel to each other shall be a very simplistic way of seeing the coagulation cascade. Now the process of haemostasis is divided into 3 phases - initiation, amplification and propagation. Initiation phase includes the normal extrinsic plus the common pathway of thrombin formation but here the thrombin formed is very limited and not sufficient. So, alongside activating factor X, factor VII-TF complex also converts factor IX to IXa of the intrinsic pathway which (in presence of factor VIII) leads to “sustained” thrombin production via the common X-Xa pathway which results in stable clot formation. This is the amplification phase. Apart from this, the thrombin formed also directly activates factor XI and further lead to self perpetuation of intrinsic pathway from factor IX onwards. This is the propagation phase. But this third phase takes less part in normal homeostatic plug formation.^11^ Fig. 2^12^

## 2 Current state of anticoagulants

**Fig. 3:**
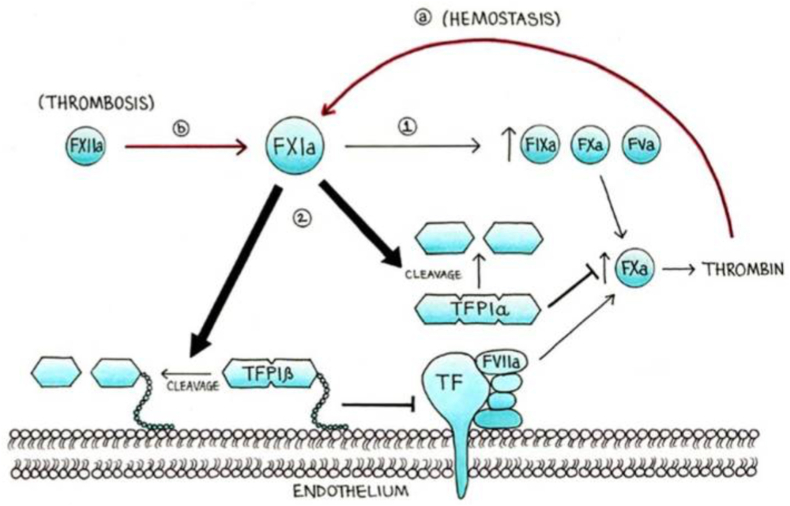
Apart from activating factor IX, X and V (step 1), activated factor XI i.e.XIa, promotes, thrombin production by triggering the extrinsic pathway which involves the tissue factor (TF) and factor VIIa (TF-FVIIa) pathway (step 2), this also includes the inactivation of Tissue Factor Plasminogen Inhibitor-α (TFPI-α) from platelets and TFPI-β from endothelial cells. This new concept also shows that in hemostasis. the main activator of factor XI is thrombin (a), whereas during thrombosis factor XI is mainly activated by factor XIIa (b).^20^.

In the field of anti-thrombosis we have three group of drugs - 1. Antiplatelets, 2. Anticoagulants and 3. Fibrinolytics. Focussing on anticoagulants, we initially had only heparin which was discovered in 1916 by McLean at Johns Hopkins university and the term was coined by Howell and Holt in 1918.^13^ Later, coumarin derivatives were discovered. They targeted both factor X and factor II. Slowly, Low Molecular Weight Heparins (LMWH) were discovered by Johnson in 1976.^14^ But the main problem with these molecules were that their pharmacodynamics was unpredictable and required regular monitoring. Then came the era of specific factor inhibitors like Bivalirudin and Argatroban which specifically inhibited factor II. But the main issue was their parenteral administration. So in 1995 Himmelsbach et al.^15^ developed an orally active, factor IIa inhibitor Dabigatran which was taken up for clinical development in 2007 (as Ximelagatran was withdrawn due to hepatotoxicity).^16^ Alongside factor Xa specific molecules like Rivaroxaban (in 2000), Betrixaban (in 2004), Apixaban (in 2007) and Endoxaban (in 2009) were also discovered and approved for clinical use by FDA in the year 2011, 2017, 2012 and 2015 respectively.^17^^,^^18^ These new drugs addressed one of the most daunting problem with heparin and coumarin derivative with regards to regular monitoring of Prothrombin Time (PT) or activated Plasma Thromboplastin Time (aPTT) levels. But then, the issue common to all these drugs was “increased bleeding tendency” and this was nothing a sort of surprise as it was well accepted that an anticoagulant would obviously have bleeding as its adverse effect. With the new concepts of extrinsic and intrinsic pathway merging at the level of factor XI^19^ and that haemostasis and thrombosis are two different phenomenon (Fig. 3),^20^ urged the scientists to discover a new way through which the thrombotic pathway could be blocked without harming the haemostatic pathway. Also, one of the main concern while using these drugs (apart from heparin) is that patients of chronic kidney diseases, which is a procoagulant condition, need to undergo dose modification and in view of this they are mostly under treated. Similarly doctors hesitate to start a DOAC (Direct acting Oral Anticoagulants) in patients with atrial fibrillation who have higher bleeding scores.

## 3 Justification for targeting factor XI and factor XII

Current arsenal of anticoagulant drugs have targeted factor II and factor X which are a part of common pathway. Though this inhibits the thrombosis but then it also inhibits the physiological haemostasis. Knowledge from the congenital deficiency of factor XI and factor XII showed that these patients were at reduced risk of ischemic stroke and venous thromboembolism (VTE) but with no increase in risk of bleeding (though findings were stronger for factor XI than XII).^21^ Bleeding only occurred when levels of factor XI decreased to less than 20 % from the normal serum level. Also, increased level of these factors made patients more vulnerable for developing VTE and ischemic stroke (stronger evidence for factor XI than XII).^22^ Animal studies showed that inhibiting factor XI or XII reduces thrombus growth, but unlike thrombus formation in rats, factor XI inhibition in primates limit thrombus formation to a greater extent than factor XII inhibition.^7^^,^^23^, ^24^, ^25^ Thus, in this race to become the Holy Grail of anticoagulation and not hampering the normal haemostasis, factor XI is ahead of factor XII and may be a better anti-thrombotic target in humans, though trials are going on at equal pace for both the novel molecules. These trials shall help to uncover the reality clarifying that inhibition of which molecule is of greater importance factor XII or factor XI. Looking at the newer concept of coagulation cascade it is evident that factor XII is not required for haemostasis but because it triggers surface-induced coagulation makes it an ideal target.^23^ Furthermore, it has also been observed that factor XII inhibition limits prekallikrein activation thus blunting inflammation.^7^ But animal and human trials with respect to factor XII inhibition show that it may serve a less important role in preventing thrombosis in humans than factor XI.^26^^,^^27^ Though this may hold true for myocardial infarction, ischemic stroke and VTE, it does not rule out the role played by factor XII in thrombosis on non-biologic surfaces like medical devices eg. pacemaker, intracardiac defibrillator, cardiac resynchronisation devices, left ventricular assist device or extracorporeal membrane oxygenation machines. So it has been proposed that, inhibiting factor XI would impede contact activation-induced thrombin generation as well as the thrombin - initiated positive feedback loop which activates factor IX using factor XIa.^28^ The Dutch RATIO (Risk of Arterial Thrombosis In relation to Oral contraceptives) study done on more than 200 young women with myocardial infarction found higher levels of proteins and factors of the intrinsic pathway associated with arterial thrombosis. Study also showed that the risk of myocardial infarction was more when kallikrein/C1-INH complexes levels were high but no such association was evident for factor XII levels.^29^ Another study done in the same population cohort showed that factor XII clotting activity was associated with decreased risk of myocardial infarction.^30^ As the science of coagulation progresses there shall be better understanding of the coagulation pathways and shall become clear on which factor to target to achieve best inhibition of thrombosis without harming haemostasis. We did a systematic analysis of all the trials that were available till the time of writing this article on various search engines and came up with following drugs which were in the pipeline as a factor XI or XII inhibitor.

## 4 Trials involving factor XI and XII inhibition

1. IONIS-FXIRx - It is an antisense oligonucleotide (ASO) of factor XI which acts by inhibiting messenger RNA (mRNA) as well as catalytic degradation of mRNA and so there is reduced hepatic synthesis of factor XI. It was an open label parallel group study involving 300 patients of unilateral total knee arthroplasty. The incidence of VTE was 27 % and 4 % in group getting (subcutaneous weekly) 200 mg or 300 mg of the study drug respectively, as compared to 30 % in enoxaparin 40 mg once daily group. 200 mg dose was non-inferior whereas 300 mg dose was superior to enoxaparin 40 mg (*p* < 0.001).^31^ Another trial with the same drug was done in 43 End Stage Renal Disease (ESRD) patients. It was a double blinded randomised controlled trial (RCT) which showed that the pharmacokinetics of the study molecule was same before and after haemodialysis. Another RCT with the same drug was done in 213 patients of ESRD. Results have not been published yet but they have been posted on pubmed clinical trial section on 20-01-2023 (NCT03358030).^32^
2. Osocimab - This was a fully humanized IgG1 monoclonal antibody against factor XIa which binds to active site of the molecule and prevents it from activating factor IX. In a randomised, open label, phase 2 non-inferiority FOXTROT trial, osocimab (0.3, 1.8 mg/kg) pre-operative or (0.3, 0.6, 1.2 or 1.8 mg/kg) post-operative, given intravenously (iv), was studied against subcutaneous enoxaparin 40 mg daily or apixaban 2.5 mg orally twice daily with primary endpoint as VTE between 10th to 13th day post-op. As half life of the drug was 30–44 days after iv administration, only single dose was used in the trial. Results showed that in knee arthroplasty patients, postoperative osocimab dose of 0.6 mg/kg, 1.2 mg/kg, and 1.8 mg/kg met criteria for non inferiority when compared with enoxaparin, and the preoperative 1.8-mg/kg dose of osocimab met criteria for superiority when compared with enoxaparin for VTE at 10th **t**o 13th days post-op.^33^ Another randomised, double blind, placebo controlled phase 2 trial, the CONVERT trial, has recently been completed in September, 2023. It studied the safety and tolerability of osocimab in 686 ESRD patients. Results have not been published yet but they have been posted on pubmed clinical trial section on 21-07-2023 (NCT04523220).^34^
3. Abelacimab - It is a monoclonal antibody against factor XI which blocks the conversion of inactive form to active form by either XIIa or IIa. An open label parallel group trial, the ANT- 005 TKA trial, single dose abelacimab (30, 75 or 150 mg), given pre-op or within 12 h post-op period, was compared against 40 mg enoxaparin given twice daily till venography was done to look for VTE in knee replacement patients. The 30 mg dose was found non inferior to enoxaparin but the 75 mg and 150 mg abelacimab dosing were superior to enoxaparin (*p* < 0.001).^35^ Another randomised phase 2 trial, the AZALEA-TIMI 71 trial, evaluated the bleeding profile of abelacimab versus rivaroxaban in 1200 patients with atrial fibrillation (AF) who were at moderate-to-high risk of stroke. It was the longest and the largest trial till date comparing factor XI inhibitors to DOACs. Trial was stopped prematurely due to overwhelming reduction in the composite of major and clinically relevant non-major bleeding in patients taking abelacimab versus those patients who were on rivaroxaban.^36^ An ongoing randomised, open label, phase 3 trial, the ASTER trial, will evaluate the effect of abelacimab (150 mg given monthly subcutaneously) as compared to apixaban (10 mg followed by 5 mg twice daily) given over a period of 6 months for VTE recurrence and bleeding in cancer associated VTE patients. Recruitment is still going on and trial is expected to be completed by October, 2024.^37^ Similar to this, MAGNOLIA trial is going on but it will compare abelacimab with dalteparin for VTE recurrence and bleeding in patients of gastrointestinal/genitourinary cancer associated VTE. It is a phase 3 trial and results are expected to be out by January, 2025.^38^
4. Milvexian - It is a small molecule, active‐site inhibitor of factor XIa with can be taken orally. In a randomised, phase 2, parallel group, open table trial, the AXIOMATIC-TKR trial, done in 1242 patients using seven different dosage of milvexian versus 40 mg Enoxaparin subcutaneous once daily it was observed that the primary outcome of VTE was less in milvexian as compared to enoxaparin group along with low risk of bleeding.^39^ On the similar lines, AXIOMATIC-SSP trial was conducted. It was a phase 2, randomised, double-blind, placebo-controlled, dose-ranging study which evaluated efficacy & safety of milvexian and the primary endpoint was composite of new symptomatic ischemic stroke and new covert cerebral infarction as detected by Magnetic Resonance Imaging at Day 90. Total of 2366 patients were evaluated after being randomised into 7 different dosing groups. A relative risk reduction of nearly 30 % was observed in symptomatic ischemic stroke in patients who were on milvexian either 25, 50 or 100 mg twice daily as compared to placebo.^40^
5. Xisomab 3G3 - It is a humanized, IgG2b monoclonal, anti factor XI antibody which blocks factor XIIa mediated factor XI activation without inhibiting FXI activation by thrombin or the procoagulant function of factor XIa.^41^ Trial was done using this novel drug in ESRD patients where factor XII activation occurs due to repeated blood exposure to artificial surfaces of haemodialysis circuit that can trigger clot formation. Heparin solves this problem to a certain extent but it is not tolerated by everyone. In this phase 2 trial, patient received iv 0.25 or 0.5 mg/kg of xisomab and researchers assessed the safety and clot-reducing efficacy of the drug as compared to placebo during heparin-free haemodialysis. Occlusive events requiring change of haemodialysis circuit were less frequent, levels of thrombin-antithrombin complexes and C- reactive protein were lower after xisomab administration when compared with data collected before dosing.^42^ Another trial was done to determine the efficacy of this drug as measured by the incidence of catheter associated thrombosis in patients of lymphoma, malignant solid neoplasm or plasma cell myeloma with a central venous catheter. Patients received xisomab the drug either iv or via catheter (single dose) within 48 h of placing the catheter. After 2 days chemotherapy was given as per the dose. After nearly 2 weeks ultrasound was done to see for catheter related thrombosis and patient was followed up for 60 days for any bleeding/thrombotic episode. Study is expected to be completed by June, 2024.^43^
6. Fesomersen - It is an antisense oligonucleotide of factor XI similar to IONIS-FXIRx which inhibits factor XI mRNA. It is also given subcutaneously weekly. This drug was compared against placebo in RE-THINc ESRD trial which was a phase 2, randomised, double-blind, placebo-controlled study evaluating the safety and pharmacokinetics of the drug in 307 ESRD patients and divided into 3 cohorts receiving 40, 80 or 120 mg of subcutaneous fesomersen or placebo every four weeks for up to 48 weeks. Primary end point was incidence of major bleeding and clinically relevant non-major bleeding (CRNMB). Results of phase 2b trial, showed decreased occurrence of dialysis circuit clotting and arteriovenous access thrombosis diminished significantly with decreasing FXI levels.^44^
7. Asundexian - It is a small molecule, reversible, oral inhibitor of factor XIa. The best thing is that its bioavailability is not affected by the first pass metabolism, formulation, food or gastric pH.^45^ This novel drug was evaluated in the PACIFIC phase II clinical trial which had three phase IIb trials under it comprising of more than 4000 patients.^46^ Main focus was to see if this drug could be a treatment for secondary prevention in patients with a non‐cardioembolic ischemic stroke, atrial fibrillation and recent myocardial infarction (PACIFIC ‐STROKE, -AF, - AMI) either as a standalone therapy, or in along with anti‐platelet therapy. PACIFIC-STROKE revealed that asundexian neither reduced the incidence of ischemic stroke nor increased the chance of major or CRNMB as compared to placebo in patients with acute, non-cardioembolic ischaemic stroke.^47^ In PACIFIC-AF trial asundexian dosage of 20 mg and 50 mg orally once daily led to lower rates of bleeding when compared to standard dosing of apixaban.^48^ For acute myocardial infarction patients, PACIFIC-AMI gave a ray of hope regarding inhibiting the thrombotic milieu without increasing the risk of bleeding.^49^ The results showed lesser rate of ischemic events without increased rate of bleeding in patients receiving asundexian. Based on these results, the OCEANIC Program (randomised, double blind, phase III trial) was done which shall evaluate asundexian in AF patients at risk of stroke and patients with acute non-cardioembolic ischemic stroke or those at high-risk transient ischemic attack (TIA). Asundexian had significantly reduced major bleeding events as compared to apixaban but it showed lower efficacy in preventing episodes of systemic embolism and stroke. Due to these reasons the trial was stopped prematurely.^50^
8. Ir-CPI - Ixodes Ricinus-Contact Phase Inhibitor is a dual inhibitor of factor XIIa and factor XIa. It is a protein expressed by the salivary glands of the tick *Ixodes ricinus* which inhibits thrombus formation without impairing haemostasis. This experimental drug is now in phase 2a under BIRCH-trial. It is a randomized, open-label, trial which will look into the safety, tolerability and efficacy of this drug in patients with spontaneous intracerebral hemorrhage. Started in July, 2023, the estimated completion date is August, 2025 (NCT05970224).^51^
9. Factor XII - antisense oligonucleotide - This novel drug decreases the hepatic synthesis of factor XII by causing catalytic degradation of mRNA responsible for its formation. Animal models have shown that anticoagulant effect of factor XII ASO treatment is sustained in both venous and arterial thrombosis and in addition to this, no prolongation of tail bleeding time was observed.^52^

10. Garadacimab - This is fully humanized IgG4 monoclonal antibody which binds to the enzymatic active site of factor XIIa and inhibit its function to activate factor XI. Animal models have shown promising results with respect to reducing carotid artery thrombotic occlusion or ECMO-circuit related thrombosis. Analysis of a phase I, first‐in‐human, randomized dose‐ escalation study for hereditary angioedema and Covid-19 showed that, there is a dose‐ dependent increase (complete inhibition of XIIa at 10 mg/kg) in aPTT with no change in prothrombin time after i.v. or s.c. administration of garadacimab but aPTT prolongation was not accompanied with increase in any bleeding tendency.^53^

Table 1 summarises all the factor XI and XII inhibitor drugs discussed in this article and Table 2 gives the summary of all the trials discussed above.

**Table 1:** Pharmacology of factor XI and XII Inhibitors Investigated in Phase 2 clinical studies.

Drug Name	Other Names	Developer	Class	Mechanism of Action	Route	Doses Tested	Time to Peak Concentration	Time to aPTT Prolongation	Elimination	Half-life	Drug–Drug Interactions	Phase 2 Studies
IONIS-FXIRx	BAY-2306001, ISIS-416858, ISIS-404071	Ionis Pharma	ASO	Degradation of FXI mRNA	Subcutaneous	200, 300 mg	≈6 h	3–12 wk	Metabolism & renal (limited)	≈2 wk	No	TKA, ESRD
Osocimab	BAY-1213790	Bayer AG	Monoclonal antibody	Inhibition of FXIa	Intravenous	0.3–1.8 mg/kg	≈1–4 h	≈2 h	Phagocytic cells & RES	30–44 d	No	TKA
Abelacimab	MAA868	Anthos Therapeutics	Monoclonal antibody	Inhibition of FXI and FXIa	IV, SC	30–150 mg	≈1 h (IV), 7–21 d (SC)	1–5 d (route dependent)	Phagocytic cells & RES	20–30 d	No	TKA
Xisomab 3G3	AB023	Aronora	Monoclonal antibody	Inhibition of FXI activation by FXIIa	Intravenous	0.25, 0.50 mg/kg	0.1–0.7 h	10–30 min	Phagocytic cells & RES	11–121 h	No	ESRD
Milvexian	BMS-986177, JNJ-70033093	Bristol-Myers Squibb	Small molecule	Inhibition of FXIa	Oral	25–200 mg	≈3 h	2–4 h	Hepatic (CYP450) & renal	11–18 h	CYP450 3A4 inhibitors	TKA, stroke
Asundexian	BAY-2433334	Bayer AG	Small molecule	Inhibition of FXIa	Oral	20, 50 mg	≈1–4 h	4–8 h	Metabolism & renal (limited)	14–21 h	No	AF, stroke, AMI
Fesomersen	–	Ionis Pharma	ASO	Degradation of FXI mRNA	SC	TBD	TBD	TBD	TBD	TBD	No	TBD
Ir-CPI	–	TBD	TBD	Inhibition of FXI	TBD	TBD	TBD	TBD	TBD	TBD	TBD	TBD
Garadacimab	CSL312	CSL Behring	Monoclonal antibody	Inhibition of FXIIa	IV, SC	75–600 mg	TBD	TBD	TBD	TBD	No	HAE, IPF

Abbreviations: AF = atrial fibrillation; AMI = acute myocardial infarction; aPTT = activated partial thromboplastin time; ASO = antisense oligonucleotide; CYP450 = cytochrome P450; ESRD = end-stage renal disease; FXI = factor XI; FXIa = activated factor XI; FXIIa = activated factor XII; HAE = Hereditary Angioedema; IPF = Idiopathic Pulmonary Fibrosis; mRNA = messenger RNA; RES = reticuloendothelial system; SC = subcutaneous; TKA = total knee arthroplasty; TBD = to be determined.

**Table 2:** Clinical trials involving factor XI and factor XII inhibition.

Trial Name	Drug	Study Design	Population	Comparator	Primary Endpoint	Status	Result
FXI-ASO TKA	IONIS-FXIRx	Open-label, parallel-group	TKA patients (*n* = 300)	Enoxaparin	VTE incidence	Completed	Reduced VTE incidence
EMERALD	IONIS-FXIRx	Double-blind RCT	ESRD patients (*n* = 213)	Placebo	Pharmacokinetics, VTE	Ongoing	TBD
FOXTROT	Osocimab	Randomized, open-label, phase 2	TKA patients	Enoxaparin, Apixaban	VTE prevention	Completed	Comparable VTE prevention to standard care
CONVERT	Osocimab	Double-blind RCT	ESRD patients (*n* = 686)	Placebo	Safety, tolerability	Ongoing	TBD
ANT-005 TKA	Abelacimab	Open-label, parallel-group	TKA patients	Enoxaparin	VTE prevention	Completed	Effective in VTE prevention
AZALEA-TIMI 71	Abelacimab	Randomized phase 2	AF patients	Rivaroxaban	Bleeding profile	Stopped early	Higher bleeding risk
ASTER	Abelacimab	Open-label phase 3	Cancer-associated VTE	Apixaban	VTE recurrence, bleeding	Ongoing	TBD
MAGNOLIA	Abelacimab	Phase 3 trial	GI/GU cancer-associated VTE	Dalteparin	VTE recurrence, bleeding	Ongoing	TBD
AXIOMATIC-TKR	Milvexian	Randomized, phase 2	TKA patients (*n* = 1242)	Enoxaparin	VTE prevention	Completed	Effective VTE prevention
AXIOMATIC-SSP	Milvexian	Phase 2, double-blind	Stroke patients	Placebo	Ischemic stroke prevention	Completed	Reduced stroke risk
PACIFIC	Asundexian	Phase 2b trials	Stroke, AF, AMI patients	Placebo, Apixaban	Bleeding, efficacy	Completed	Lower bleeding risk than standard care
BIRCH	Ir-CPI	Randomized, open-label	Intracerebral hemorrhage patients	–	Safety, tolerability	Ongoing	TBD
RE-THINc ESRD	Fesomersen	Double-blind, placebo-controlled	ESRD patients (*n* = 307)	Placebo	Bleeding risk, VTE prevention	Completed	Reduced bleeding risk
OCEANIC	Asundexian	Phase 3 trial	AF, Stroke, TIA patients	Placebo	Stroke prevention	Ongoing	TBD
Phase 1 study	Garadacimab	Dose-escalation, first-in-human	Hereditary angioedema, COVID-19	–	aPTT prolongation, safety	Completed	Prolonged aPTT, well tolerated

Abbreviations used - AF = Atrial fibrillation, AMI = Acute myocardial infarction, aPTT = Activated partial thromboplastin time, ESRD = End-stage renal disease, FXI = Factor XI, FXII = Factor XII, GI/GU = Gastrointestinal/Genitourinary, RCT = Randomized controlled trial, SC = Subcutaneous, TBD = To be determined, TIA = Transient ischemic attack, TKA = Total knee arthroplasty, VTE = Venous thromboembolism.

## 5 Conclusion and the future path

The development of Factor XII and Factor XI inhibitors represents a promising advancement for patients whose anticoagulation needs remain unmet by direct oral anticoagulants (DOACs) or low-molecular-weight heparins (LMWH). These novel agents hold significant potential for populations requiring enhanced coagulation control, including patients with end-stage renal disease (ESRD), atrial fibrillation (AF), cancer-associated thrombosis, and those undergoing post-arthroplasty management.

The evolution of anticoagulant therapy has progressed remarkably from its origins in the 17th century to the refined agents available today. However, a persistent challenge remains—the inherent risk of bleeding, which often leads to under-dosing and suboptimal anticoagulation. Current research aims to decouple the processes of thrombosis and hemostasis, a paradigm shift that underpins the therapeutic rationale for Factor XII and Factor XI inhibitors.

Data from completed and ongoing Phase 2 and Phase 3 trials have demonstrated promising efficacy and safety profiles, reinforcing the potential integration of these agents into the anticoagulation armamentarium. However, the results of large-scale Phase 3 trials are eagerly awaited. Key considerations in the development of these drugs include cost-effectiveness and the concurrent development of specific antidotes to mitigate bleeding risks. Given that many of these agents are biologics or gene-based therapies, pricing remains a critical factor. Notably, among monoclonal antibodies approved by the U.S. Food and Drug Administration (FDA) between 1997 and 2016, the average annual cost was approximately $100,000 per patient.^54^

The continued advancement of Factor XII and Factor XI inhibitors holds the potential to reshape anticoagulation strategies, providing safer and more targeted therapeutic options for high-risk patient populations.

## Declaration of competing interest

The authors declare that they have no known competing financial interests or personal relationships that could have appeared to influence the work reported in this paper.

## Abbreviations used

i. aPTT -: activated Plasma Thromboplastin Time
ii. ASO -: Anti Sense Oliginucleotide
iii. DOAC -: Direct acting Oral Anticoagulants
iv. ESRD -: End Stage Renal Disease
v. LMWH -: Low Molecular Weight Heparin
vi. PT -: Prothrombin Time
vii. RCT -: Randomised Control Trial
viii. TF -: Tissue Factor
ix. VTE -: Venous Thromboembolism
